# Strong Spatial Influence on Colonization Rates in a Pioneer Zooplankton Metacommunity

**DOI:** 10.1371/journal.pone.0040205

**Published:** 2012-07-06

**Authors:** Dagmar Frisch, Karl Cottenie, Anna Badosa, Andy J. Green

**Affiliations:** 1 University of Oklahoma Biological Station, University of Oklahoma, Kingston, Oklahoma, United States of America; 2 Department of Integrative Biology, University of Guelph, Guelph, Canada; 3 Department of Wetland Ecology, Doñana Biological Station (EBD-CSIC), Sevilla, Spain; University of San Diego, United States of America

## Abstract

The magnitude of community-wide dispersal is central to metacommunity models, yet dispersal is notoriously difficult to quantify in passive and cryptic dispersers such as many freshwater invertebrates. By overcoming the problem of quantifying dispersal rates, colonization rates into new habitats can provide a useful estimate of the magnitude of effective dispersal. Here we study the influence of spatial and local processes on colonization rates into new ponds that indicate differential dispersal limitation of major zooplankton taxa, with important implications for metacommunity dynamics. We identify regional and local factors that affect zooplankton colonization rates and spatial patterns in a large-scale experimental system. Our study differs from others in the unique setup of the experimental pond area by which we were able to test spatial and environmental variables at a large spatial scale. We quantified colonization rates separately for the Copepoda, Cladocera and Rotifera from samples collected over a period of 21 months in 48 newly constructed temporary ponds of 0.18–2.95 ha distributed in a restored wetland area of 2,700 ha in Doñana National Park, Southern Spain. Species richness upon initial sampling of new ponds was about one third of that in reference ponds, although the rate of detection of new species from thereon were not significantly different, probably owing to high turnover in the dynamic, temporary reference ponds. Environmental heterogeneity had no detectable effect on colonization rates in new ponds. In contrast, connectivity, space (based on latitude and longitude) and surface area were key determinants of colonization rates for copepods and cladocerans. This suggests dispersal limitation in cladocerans and copepods, but not in rotifers, possibly due to differences in propagule size and abundance.

## Introduction

According to neutral theories such as the theory of island biogeography [1] and the unified neutral theory of biodiversity and biogeography [2], both habitat size and spatial isolation (distance from source population) are important determinants of biodiversity. A variety of studies across a broad range of taxa show that larger islands (habitats) collect a higher amount of species (a target effect), and suggest that distance generally limits dispersal and thus also affects community structure [3], [4], [5], [6], and others. Additionally, species-area relationships and spatial patterns of species diversity and community dissimilarity depend on both the strength of dispersal and establishment capacity [7]. On the other hand, niche theories predict higher species richness in environmentally heterogeneous habitats, and predict communities to be mainly structured by local factors where colonization is restricted by niche requirements of species [8], [9]. Combining Hutchinson’s niche concept with metapopulation theory and source-sink theory could provide a strong theoretical basis for the understanding of species distributions [10].

While many studies account for neutral and niche processes separately, the metacommunity framework combines aspects of both concepts [11], involving four main models with varying degrees of spatial (regional) and environmental (local) influence. These models were empirically tested in a meta-analysis of 158 metacommunity data sets by [12] who concluded that 44% of the studied metacommunities were structured solely by environmental factors (the species sorting model), while spatial patterns best explained the community structure (the neutral model or patch dynamics) in 8%, and both spatial and environmental components significantly influenced community structure in 29% of metacommunities (i.e. a combination of the species sorting model with either mass effects or dispersal limitation, see [13]).

The magnitude of community-wide dispersal is central to these metacommunity models [11]. Dispersal is notoriously difficult to quantify in passive and cryptic dispersers such as many freshwater invertebrates [14]. Much recent empirical research on dispersal of freshwater zooplankton has used colonization, or the arrival of new species per time interval, as a proxy [15], [16], [17]. Dispersal rates determine but do not equal colonization rates, since establishment might be hampered by local conditions such as unsuitable habitat, biotic interactions [18], [19] or Allee effects [20]. Since only successful colonization events can be observed, colonization rates tend to underestimate dispersal. In the case of zooplankton, this problem can be reduced by measuring colonization rates in new habitats, where they are likely to be more accurate estimates of effective dispersal rates than in established communities, given that biotic interactions are overshadowed by random colonization events [21], Allee effects and stochastic extinctions [22] that determine the species present for those interactions. Environmental filtering can be more important than biotic interactions in temporary ponds [23], where densities of zooplankton predators such as macroinvertebrates, fish or amphibians are lower, since they are strongly dispersal limited [15] and generally lack dormant stages. Phytoplankton, protists and bacterioplankton are fast and ubiquitous dispersers [24], and provide resources essential for the establishment of zooplankton arriving via dispersal. Environmental filtering may be important in new habitats, e.g. since many zooplankton species are adapted to salinities, and some are highly dependent on aquatic macrophytes that take longer to establish [25], [26].

Freshwater habitats usually have well defined boundaries, embedded in a matrix of uninhabitable terrestrial areas across which aquatic organisms must disperse, and are therefore good testing grounds for metacommunity studies. Empirical evidence from such habitats for colonization by zooplankton has been inconsistent, likely due to the differences of the degree of habitat isolation among these studies [21]. Jenkins and Buikema [27] and Jenkins and Underwood [28] found low colonization rates and assumed strong dispersal limitation in their systems. In their review on dispersal of freshwater invertebrates, Bohonak and Jenkins [15] maintain that “*although individuals of various species certainly disperse on long time scales, we interpret the currently available evidence as rejecting the notion that overland dispersal in most freshwater taxa is frequent and widespread on relatively short time scales*”. In contrast, the high colonization rates found both in experimental mesocosm studies [17] and in new ponds [21] dispute the claim of strong dispersal limitation in freshwater zooplankton. Louette and De Meester [21] found indications of influence of spatial isolation on dispersal of cladocerans, but no indication for local control during the first 15 months of pond existence. However, none of these zooplankton studies systematically examined the relative importance of spatial versus environmental factors on colonization rates of different zooplankton taxa within pioneer communities.

Dispersal rates (i.e. number of dispersing individuals per time unit) are largely determined by dispersal capacity. In zooplankton, dispersal capacity and hence colonization rates are likely to be strongly influenced by their respective dispersal vectors [29], especially mammals, birds, insects, wind and rain [16], [17], [30], [31], [32]. Cyclopoid copepods and rotifers are often the first colonists, while cladocerans arrive at a later stage [16], [17], [31], possibly due to differences in dispersal capacity. Priority effects are also important, with the initial assemblage partly determines future development of communities [33], [34].

Here, we present a systematic analysis of the relative influence of spatial and environmental factors on colonization rates of zooplankton during the early stages of community assemblage. A set of ponds of different size and spatial arrangement was constructed within a large restoration project, providing a rare possibility of following early colonization of zooplankton at spatial and temporal scales relevant for natural systems. We quantified colonization rates (rates of cumulative increase in species number) separately for cladocerans, copepods and rotifers. These groups were chosen to represent basic life history traits which can influence dispersal and colonization capacity; copepods reproduce sexually while cladocerans and rotifers reproduce mostly parthenogenetically, and all three differ generally in their size of dispersal stages (rotifers have the smallest, and cladocerans the largest). We repeatedly sampled 48 newly constructed temporary ponds for the first 21 months of their existence, equalling a cumulative total of four to six months of hydroperiod. The link between colonization rates as estimates of effective dispersal will be less biased and the processes involved in the colonization of new habitats can be better understood when species accrual in these new sites is compared with that in pre-established, environmentally similar reference sites within the same region [35], [22]. We therefore simultaneously sampled the regional species pool in neighboring, natural temporary reference sites. The rate of species accumulation may result from several processes: arrival of new species, natural species turnover caused by seasonal and yearly variation, and an increased sample size caused by multiple sampling of the same pond. The accumulation rates of zooplankton species in established ponds probably mostly reflect the two latter processes. By contrasting accumulation curves in new versus established pools, we can dissociate colonization from these other two processes. For the purpose of this study, we define “colonization” as a series of steps including dispersal from a source, followed by arrival at the new habitat patch and establishment, including reproduction and population increase up to a detectable density.

We computed two descriptors to study the colonization process: initial species richness at first sampling, and colonization rates as quantified by the accumulation of species throughout the hydroperiods. We predicted that new (experimental) ponds would have lower initial species richness and higher colonization rates compared to richness and recolonization rates in the existing (reference) ponds.

In a second step, we used variation partitioning and Redundancy Analysis (RDA) to identify the contribution of spatial and environmental factors to colonization rates into new ponds in each of the taxonomic groups. We predicted that (1) in the case of dispersal limitation, colonization into new habitats will be related to spatial/hydrological connectivity, pond surface area and/or colonization distance, but not to environmental heterogeneity, and (2) that zooplankton taxa would be differentially dispersal-limited, related to differences in their size and abundance.

## Materials and Methods

### Experimental Ponds and Reference Ponds

**Figure 1:**
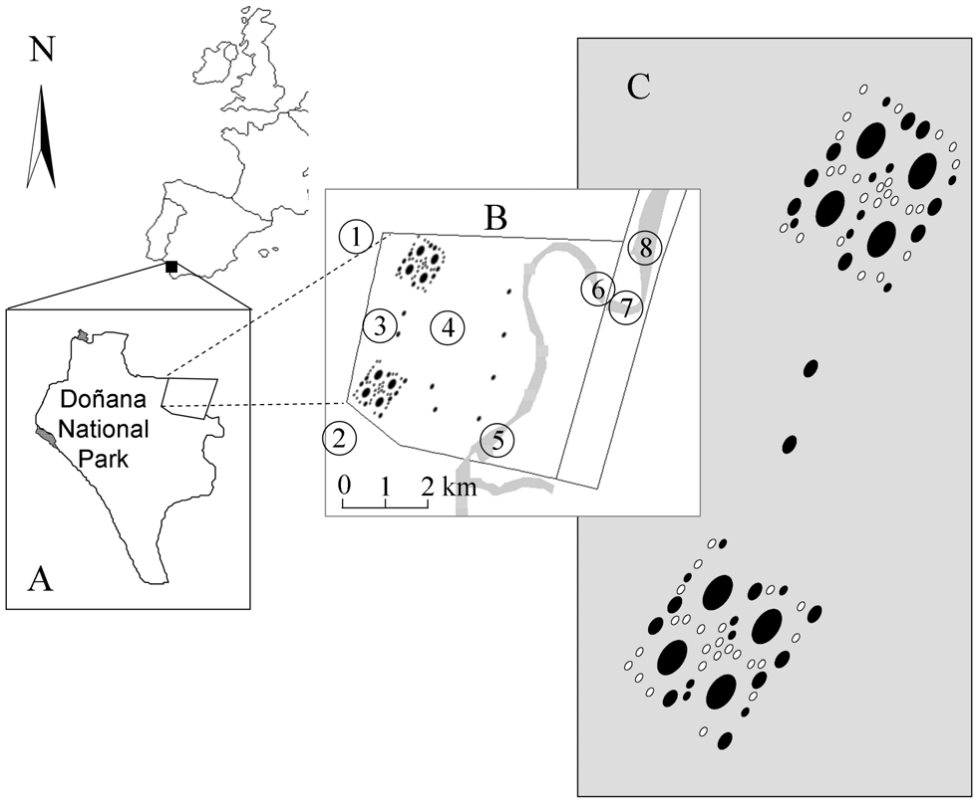
Map of pond area. Location of Caracoles estate within the Doñana National Park in Southern Spain (A). Map detail with scale bar showing the location of the experimental ponds (black filled circles) and the reference ponds (numbers 1–8) (B). Map detail showing the spatial arrangement of all experimental ponds (all circles), the ones sampled for this study are shown as black filled circles (C).

The experimental ponds are located within Caracoles estate (37°07’N, 6°31’W, Fig. 1) in Doñana National Park (Southwest Spain). This area of former temporary marsh (2,700 ha) was transformed to agricultural land in the 1970 s, drained and intensively cultivated with cereals until 2004. In 2004, Caracoles estate was added to the National Park area as part of a large restoration effort to compensate for the loss of most of the natural temporary marshes in the Guadalquivir delta over the 20^th^ century (the "Doñana 2005"project, [36]). For more details on the Caracoles estate and the surrounding natural marshlands see [25], [35], [37], [31]. As part of the restoration, a set of experimental ponds was excavated in Caracoles estate (Fig. 1 and [35]) between 2004 and 2005. This experimental pond area (see Fig. 1 for spatial setup) contains 96 elliptically-shaped temporary ponds of three different surface areas (with a long axis of 250 m, 125 m and 60 m in 8, 24 and 64 ponds, respectively) and two excavation depths (30 and 60 cm). Note, the actual water depths of the ponds varied during sampling periods, related to rainfall and occasional overspill (see below). All ponds fill by rainwater and are not connected to rivers or streams. Eight medium sized ponds are spatially isolated and scattered across the area, while all others are clustered in two groups (see black circles in Fig. 1b and c). A subset of the ponds was built on former drainage ditches which during restoration were filled with top soil, so these ponds may have received propagules formerly present in the ditches or in sediment used as filling material. However, sediment cores studied in a hatching experiment found no evidence of a preexisting propagule bank in these experimental ponds [31].

Eight temporary, shallow water bodies in the immediate neighborhood (Fig. 1, max. distance 3 km) were included as reference sites in the present study: two large natural temporary ponds (Lucio del Lobo, Lucio de Mari Lopez, sites 1 and 2 in Fig. 1, respectively), and two natural ponds that form in ground depressions within the Caracoles estate (sites 3 and 4 in Fig. 1), two ponds in the bed of a former stream that ran through the Caracoles estate but has been isolated from the upstream section by dyke construction since the 1960s (sites 5 and 6 in Fig. 1), and two sites in a similar stream east and northeast of the estate (Entre Muros, sites 7 and 8 in Fig. 1). The four reference sites within the Caracoles estate existed as temporary ponds even before the restoration project [25], although their hydroperiods were generally shorter. Choice of reference ponds was limited because the Caracoles estate is largely surrounded by drained farmland to the North and East, and a continuous and inaccessible marshland to the south and west [38], [39]. All reference sites (“reference ponds” from here on) were fed by rainwater and had hydroperiods, depths and clay soils similar to the new ponds. There was extensive overlap in the water chemistry between reference and new ponds (Table S1). Reference sites had both emergent and submerged vegetation, and fish and amphibians were present in some of the reference ponds outside the Caracoles estate [25], [35]. During the present study in the new ponds, submerged plants began to colonize at low density [35] but emergent aquatic plants were absent. Fish and amphibians were also absent and waterbirds known to feed on zooplankton (e.g. greater flamingo or shoveller) were rare in the new ponds (census data available in http://www-rbd.ebd.csic.es/Seguimiento/mediobiologico/aveshumedales/anteriores.html).

All necessary permits were obtained for the described field studies. Permits required to enter Doñana National Park were issued by the Consejería de Medioambiente, Junta de Andalucia.

### Sampling and Faunal Analysis

Sampling took place monthly between pond creation and the end of 2006 when ponds carried water (March 2005, February, March, April, May and November 2006). We chose a subset of new ponds to be sampled on a regular basis for this and future studies, which included all large ponds (n = 8), all medium ponds (n = 16, of which n = 8 isolated and n = 8 within the two pond clusters) and 16 small ponds (eight chosen randomly in each cluster) to represent all three size classes. In March 2005, only six of these ponds had a small amount of water for a brief period of time [31]. The following dry period lasted until February 2006, when all new and reference ponds were filled with rainwater after heavy precipitation. The hydroperiod lasted until late May for 43 of the 48 sampled ponds whereas the remaining five ponds dried out in the first half of June. After heavy rainfall in November 2006, when all ponds refilled with rainwater, the southern pond cluster was inaccessible due to flooding of access routes. The number of ponds sampled in every size category and month is listed in Table 1. In some cases, ponds were interconnected by shallow flooded areas (up to ∼10 cm deep) during times of peak inundation, and became parts of larger waterbodies (Figure S1, S2).

**Table 1:** Number of experimental ponds with different diameters (60, 125, and 250 m) and spatial grouping (isolated/in a cluster) sampled in every month.

Pond	*dry period*	3/05	*dry period*	2/06	3/06	4/06	5/06	*dry period*	11/06
Exp-60 m		4		16	–	16	–		8
Exp-125 m, cluster		2		16	16	16	7		8
Exp-125 m, isolated		–		8	8	8	6		8
Exp-250 m		1		8	–	8	–		4
Reference ponds		–		6	2	8	–		6

Dry periods immediately preceded hydroperiods in March 2005, February 2006 and November 2006, and immediately followed hydroperiods in March 2005 and May 2006. Detailed results for samples in March 2005 and April 2006 were published in [31] and [35]. Sample months are referred to as month/year (e.g. 3/05 stands for March 2005).

Ponds were sampled by collecting subsamples with a 500 ml plastic jug from various randomly chosen points to take into account the patchy zooplankton distribution in the water column. In March 2005, total sample volume was maximally 10 L due to very small amounts of water in the ponds (for details see [31]). In 2006, about 40 subsamples were collected in each pond, and combined to a total volume of 20 L. The water was filtered through a 64 µm nylon mesh and zooplankton preserved in 70% EtOH. To avoid cross-contamination between ponds, we thoroughly cleaned the sampling equipment with tap water after sampling each pond. Plastic bags protecting our boots from contamination with sediment were changed before entering each pond. Samples in a given month were usually collected within 10 days. On each sample date we recorded water temperature, dissolved oxygen (DO), pH and conductivity *in situ*. Additionally, we obtained average water depth from five points between shore and centre of a given pond. A MANOVA performed on these variables using pond type as independent variable was significant (Wilks test  = 0.559, F_(7,182)_ = 20.49, p<0.001), although there were no significant differences in temperature, pH, conductivity or depth between experimental ponds and reference ponds. Experimental and reference ponds differed in dissolved oxygen and pond size (see Table S1 for details), despite considerable overlap between pond types.

The percentage of the pond basin that was inundated was estimated visually, and hydrological connections to other ponds or flooded areas recorded.

Copepods, cladocerans and larger rotifers (>64 µm) in each sample were mostly identified to species level (Table S2), using Dussart [40], [41], Einsle [42], Alonso [43] and Koste [44]. Species richness was identified by counting subsamples representing at least 1/16th of the total sample, and together contained at least 200 individuals of the most frequent taxon. In addition, the entire sample was screened for rare taxa not recorded in the subsample. For a complete species list with frequency in experimental and reference ponds during each sample month see Table S2.

### Estimation of Colonization Rates

To obtain colonization rates, we calculated cumulative species richness per new or reference pond for all sample dates (i.e. total combined number of species recorded until and including a given sample date), and regressed this against the number of inundation days for each sampling date. Colonization rates were calculated for the number of days a given pond was inundated (inundation days, see Fig. S3, S4, S4, S6). In addition, we added a standardized time period of 15 days before the first sample date and after the last sample date for each inundation period (2005 and 2006) to account for the period that a given pond was still inundated before the first day or after the last day of sampling.

The two parameters of this linear regression measure two different aspects of the colonization process. The intercept estimates initial species richness on the first sampling date, and the slope of this relationship equals colonization rate. The advantage of using these regression values as proxies for colonization rates is that they provide a flexible way to measure and compare colonization rates among ponds with different hydroperiods. We used the parameters from these regressions without changing the non-significant parameters to zero. Since we computed cumulative species richness as the dependent variable, these values will by default increase as these ponds mature, so that slopes must be positive. The questions of interest are whether the slopes differ between new and reference ponds, or between taxonomic groups.

### Comparisons between Taxonomic Groups and Experimental versus Reference Pools

To compare the two colonization descriptors (intercept: initial species richness, slope: colonization rates), we tested for an interaction effect between experimental condition (new versus reference pond) and taxonomic group (rotifers, copepods, cladocerans) on the intercept with a 2-way repeated measures ANOVA, with pond identity as repeated measures.

### Variation Partitioning

To assess which variables determine the two colonization descriptors for each of the major zooplankton groups, we restricted the analyses to the experimental ponds, and decomposed the variation in the intercepts and slopes for each experimental pond into variation that can be explained by four variable groups: environmental heterogeneity, pond morphometry (average area and volume of each pond), a set of connectivity measures, and “space” (see below) using partial RDA.

We computed environmental heterogeneity using three steps: 1) we obtained PCA coordinates from temperature, pH, DO and conductivity for all experimental ponds over all time steps (for a summary of environmental factors see Table S1), 2) we extracted the first two principal components explaining 54% of variation, and 3) computed the variation in the first two principal component scores over time per pond as the measure of temporal environmental heterogeneity. PCA allowed us to compare the different variables on the same scale, to reduce the number of variables, and to extract the major axes of variation, which are the most relevant when expressing environmental heterogeneity. We only used the first two axes because the amount of variation explained by the 3^rd^ axis was less than 20%, i.e. what can be expected under random variation [45].

Average area and volume were estimated individually for each pond from the flooded area of the pond basin and from depth measurements averaged over all sampling dates.

The set of connectivity measures was selected as a way of analyzing colonization distance and included the positive connectivity measures “spatial isolation” (within or outside of a pond cluster, with the rationale being that being inside a cluster with higher density of ponds in the neighborhood should increase the chances of colonization), “ditches” (whether or not constructed over a former drainage ditch), “pond connectivity” (number of experimental ponds or other waterbodies such as flooded grassland, puddles or small ditches, that a given pond was hydrologically connected to during peak flooding events), “degree of connection” (containing four classes reflecting the size of the flooded area to which a given pond was hydrologically connected: 0 =  not connected, 1 =  weak connection to small flooded area just outside pond, 2 =  connected to shallow roadside ditch, 3 =  connected to large flooded area, Fig. S1,S2), and as a negative connectivity measure “colonization distance” (geographic distance to the nearest reference pond).

For the variable “space”, we computed PCNM (principal coordinates of neighbour matrices) based on the latitudinal-longitudinal coordinates [46]. This method, which determines the different spatial scales in the geographic locations of the ponds, was used to detect the presence of significant colonization patterns at different spatial scales, from large (e.g. North-South or East-West), to small (spatial patterns within a smaller subset of clustered ponds). These PCNM axes are similar to the more familiar practice of creating 3^rd^ degree polynomials of X–Y coordinates, but they provide a more flexible way of modeling potential spatial patterns [47].

We used the variation decomposition method outlined by Legendre and Legendre [48] and Cottenie [12], updated with the unbiased estimates [49] using partial Redundancy Analysis (RDA). We used this approach because this allowed us to (1) compute the amount of variation and associated significances of the four groups of explanatory variables both by themselves and after eliminating the effects of the three other groups of explanatory variables, (2) use the flexible method of significance testing by permutation [48] instead of relying on assumptions of normality and continuous variation, (3) present the results of all variables in a triplot, and (4) determine the effect on both aspects of colonization (initial species richness and colonization rate) at the same time. For instance, we computed the amount of variation in intercepts and slopes per pond explained by environmental heterogeneity (variation in PC1 and PC2) after removing the effects of all other variables to compute the so-called “pure” environmental effects. Removing an effect in RDA is the multivariate analogue to removing the effect of a predictor variable in a multiple regression.

## Results

### Initial Species Richness

**Figure 2:**
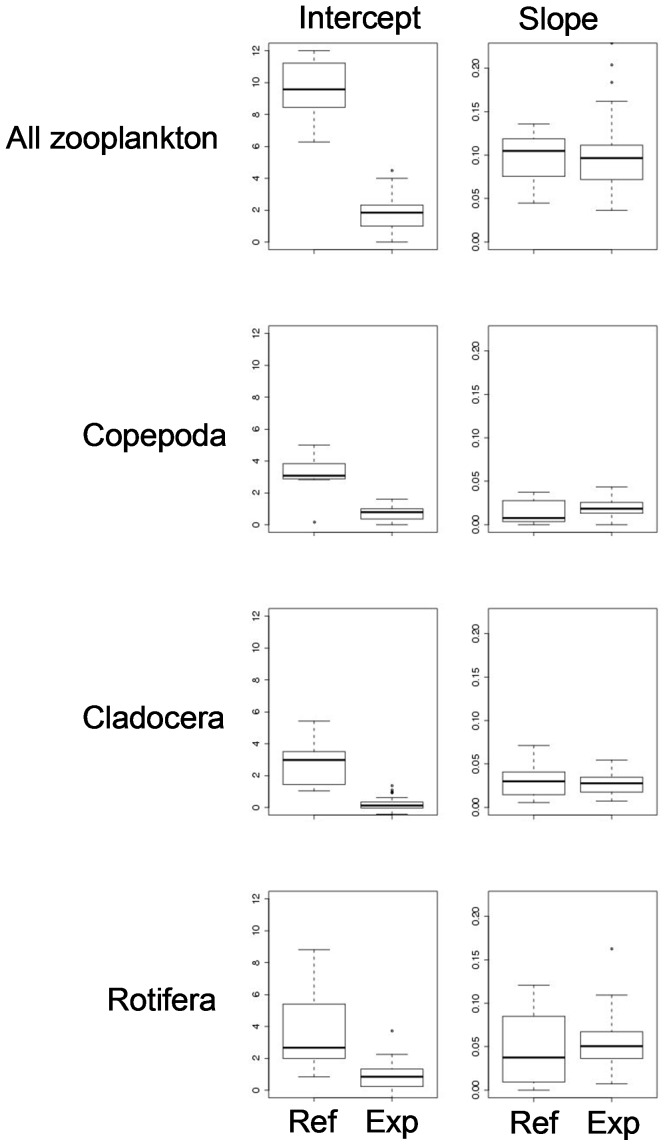
Initial species richness (intercept, left hand column) and colonization rates (slope, right hand column). The replicate units for these boxplots are the intercept and slope estimates for each pond (see text for more details). Parameters are shown for all zooplankton taxa combined (all zooplankton), and separately for copepods, cladocerans and rotifers and for reference ponds (Ref) and new ponds (Exp).

Intercepts (Fig. 2, left column) estimate the initial species richness at the beginning of the study, i.e. the number of species in the first month of each pond’s hydroperiod. The values obtained separately for the three taxa copepods, cladocerans and rotifers were on average about three times higher in reference ponds than in new ponds, but differed between taxa (2-way ANOVA, new vs. reference ponds: F_(1, 54)_ = 253, p<0.001; taxonomic group: F_(2,108)_ = 8.9, p<0.001). There was no significant interaction between pond type and taxonomic group (F_(2,108)_ = 0,41, p = 0.66).

The results of the variation partitioning analysis for experimental ponds indicated a weak and insignificant effect of area/volume and space on the intercept (results not shown). Variation partitioning analysis indicated that initial species richness in new ponds for the different subsets (cladocerans, copepods, and rotifers) had no significant relationships with any of the variables studied.

### Colonization Rates

**Figure 3:**
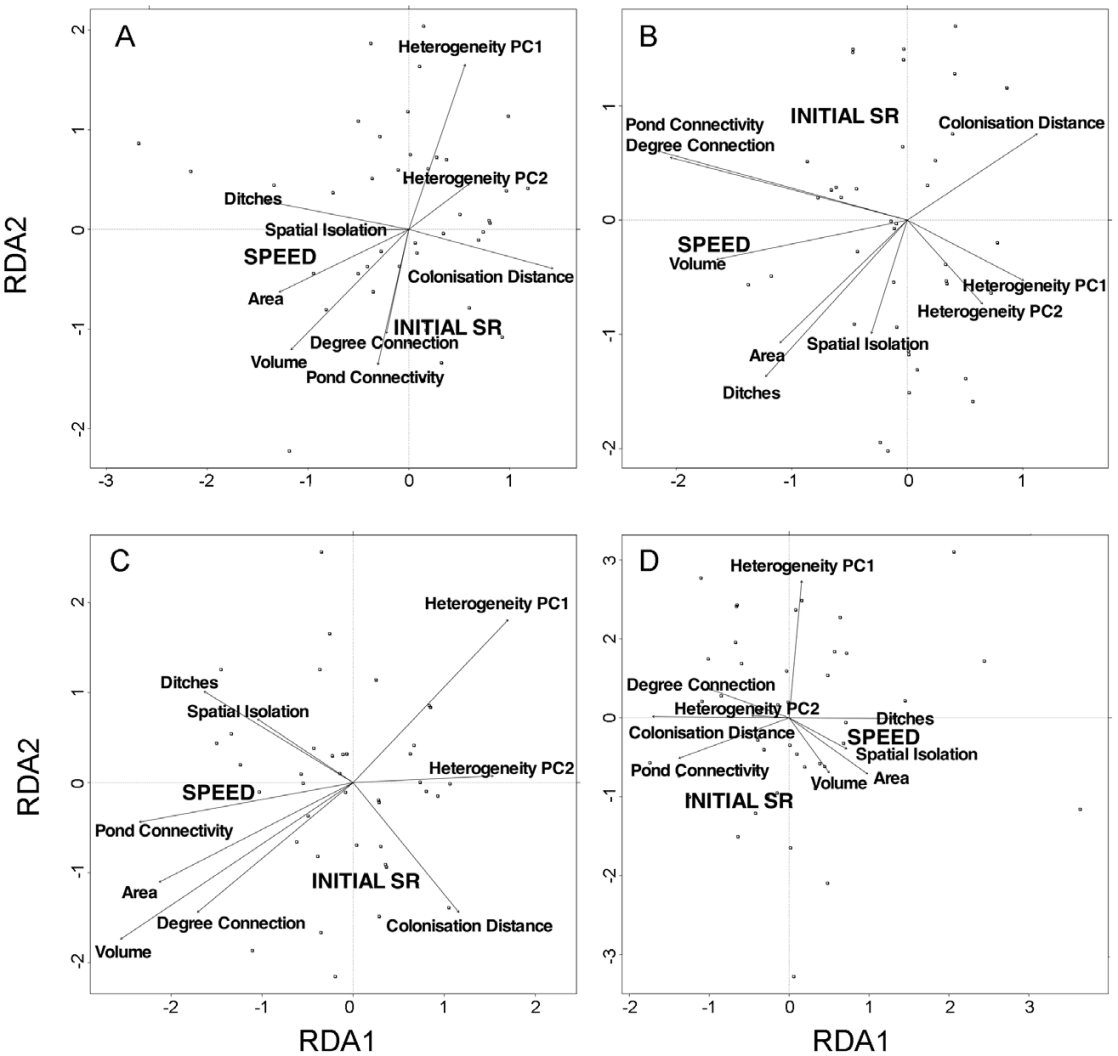
Triplots of redundancy analysis of initial species richness (Initial SR) and colonization rates in all experimental ponds studied. The triplot shows 1) the scores (locations) of these dependent variables, their relation to 2) the explanatory variables, and 3) the points that represent all experimental ponds sampled. Results are shown for all zooplankton (A), copepods (B), cladocerans (C) and rotifers (D). Explanatory variables included in the RDA are the variation in the first two principal component scores (PC1 and PC2) over time per pond that measure environmental heterogeneity; pond morphometry variables (area, volume) and connectivity measures (pond connectivity, degree of connection, ditches and colonization distance). Points represent all new ponds sampled.

Slopes (Fig. 2 right column, Fig. 3) represent colonization rates (number of species per day, see graphs in Fig. S3, S4, S5, S6). There was no significant difference in slopes between new ponds and reference sites (F_(1, 54)_ = 0.027, p = 0.86), but the effect of taxonomic group was significant (F_(2,108)_ = 49.79, p<0.001). There was no significant interaction between pond type and zooplankton group (F_(2,108)_ = 0.33, p = 0.72). New ponds were colonized at a mean rate of 0.09 (total zooplankton), 0.05 (rotifers), 0.028 (cladocerans), and 0.015 (copepods) species per day.

In the variation partitioning analysis, the RDA shows for all three taxa that environmental heterogeneity was strongly negatively related to pond connectivity and area/volume, indicating low environmental heterogeneity of the larger and deeper ponds and those overspilling into neighboring ponds or other areas (Fig. 3). The variables “spatial isolation” and “ditches” were usually positively correlated, because all ponds located outside the two pond clusters were built on a ditch. However, both these variables were unrelated to the measures used to describe hydrological connectivity.

Environmental heterogeneity did not explain a significant amount of variation in the colonization rates of any of the taxa, while “pure” effects of connectivity measures and space significantly explained slopes of copepods and cladocerans, but not of rotifers (Table 2). RDA showed that copepod colonization rates had a negative relationship with colonization distance, indicating that ponds closer to a reference pond were more readily colonized than those further away (Fig. 3b). For cladocerans this relationship was also significantly negative, but much weaker (Fig. 3c). Together, “pure” effects of space and connectivity measures explained 52% and 48% of the variance observed in copepods and cladocerans, respectively (Table 2). Both copepod and cladoceran colonization rates were positively related to pond connectivity, which can also be interpreted as another measure for surface area of a pond, since when various ponds connect to each other or to adjacent inundated areas, the total combined surface also increases. “Pure” area/volume effects were only significant for cladocerans (Table 2) and were positively related to colonization rates, explaining an additional 12% of the variation. In the RDA graphs of cladocerans (Fig. 3c), slope is positively correlated to all connectivity measures, and negatively to colonization distance, but most strongly to habitat area/volume. Colonization rates of rotifers were not significantly related to any variables in the variation partitioning analysis.

**Table 2:** Factors explaining colonization rate throughout the entire sampling period for all three zooplankton taxa combined, and separately for each taxonomic group, as determined from RDA results for slopes.

	Zooplankton		Copepods		Cladocerans		Rotifers	
	adj. r^2^	*p*	adj. r^2^	*p*	adj. r^2^	*p*	adj. r^2^	*p*
*Whole model*	0.396	0.04	0.650	0.002	0.573	0.003	–	n.s.
*Pure effects*								
Environmental heterogeneity	–	n.s.	–	n.s.	–	n.s.	–	n.s.
Area/Volume	–	n.s.	–	n.s.	0.122	0.024	–	n.s.
Connectivity measures	–	n.s.	0.301	0.004	0.233	0.017	–	n.s.
Space (PCNM)	–	n.s.	0.216	0.027	0.251	0.033	–	n.s.

Out of the potential spatial patterns (see Fig. S7), copepod colonization rates were significantly related to the first PCNM axis that contrast the northern versus the southern cluster of experimental pools after a forward selection procedure [50]. Cladoceran colonization rates were significantly related to the ninth PCNM axis that identifies some more localized differences within especially the southern cluster (see Fig. S7).

## Discussion

This study took advantage of a large restoration project, which offered a unique opportunity to study the early stages of zooplankton community assemblage by experimentally manipulating the factors influencing colonization rates, and simultaneously considering spatial and environmental factors. The results presented here increase our understanding of spatial colonization patterns at a unique, large experimental scale similar to that of natural systems.

One key finding of the present study was that, at this early stage of community assemblage, there was no indication that environmental heterogeneity (as measured here) was related to colonization rates of the studied taxa, while connectivity measures, space and pond area were important determinants of colonization rates into new ponds, but not equally for all taxa. However, we may have missed some important environmental variables in our study (see below), and it is possible that the effects of e.g. “pure space” are in fact owing to some unmeasured environmental factor that is correlated with space.

Another key finding is that our slope estimates of colonization rates based on cumulative species richness differed between zooplankton taxa (with rotifers being fastest and copepods slowest), but not between new ponds and reference sites. We would have expected slopes to be higher in new ponds than reference sites, given that mature zooplankton communities are more likely to be saturated [51]. However, our temporary reference sites lie within a highly dynamic Mediterranean system where turnover of zooplankton species is high [52]. Many zooplankton taxa not recorded in our reference sites in April 2006 were present in April 2007, and vice versa [31]. Furthermore, the smaller number of reference sites reduced the statistical power of tests comparing them with new ponds.

As expected, the new ponds were mostly uninhabited at the beginning of the study, while initial species richness of reference ponds was three times higher. This finding corroborates the results of a hatching study, which suggested near absence of propagule banks in new ponds [31]. In the new ponds, regression slopes can therefore be interpreted as estimates of colonization rates (new species arriving from outside sources) whereas in the reference ponds, which have well-established *in-situ* propagule banks with more than 5000 zooplankton hatching per m^2^ [31], these rates may to a large extent represent re-colonization from propagule banks. However, many of the new ponds overlay former drainage ditches previously filled with the topsoil from surrounding areas. We cannot rule out the possibility that this material or the ditches themselves contained zooplankton dormant stages/eggs, and thus may have artificially increased colonization rates.

Copepods, cladocerans and rotifers all rapidly colonized new ponds during the first months of inundation. Cladoceran colonization rates in the present study were about twice as high as those of Louette and De Meester [21], who found an average rate of 4.9 species in 15 months whereas we found on average four cladoceran species within the first six months of inundation. Copepod and rotifer colonization rates were similar to those observed by Cohen and Shurin [17] but higher than Jenkins and coauthors [27], [28]. Because they are obligate sexuals, copepods may show delayed colonization due to a stronger Allee effect compared to cyclical parthenogens, such as cladocerans and rotifers [20]. Although various studies have shown that cyclopoid copepods are often the first colonizers [16], [31], this might only pertain to a few species with outstanding dispersal capacity, while the colonization rates for the entire group are relatively slower.

Our results likely underestimated actual dispersal to some extent, because the possibility remains that environmental conditions or other factors precluded the establishment of dispersing individuals in some cases (e.g. see [22] for the need for dispersers to reach a critical density to enable establishment). However, since all the new ponds were environmentally more similar to each other (e.g. non-existent to small egg bank, no macrophytes, similar fishless food-web, same history) in comparison to the reference sites, environmental filtering is less likely to have influenced colonization rates. We measured variables such as salinity, pH and depth which have important influences on the structure of zooplankton communities in Mediterranean temporary wetlands [35], [26]. Nevertheless, we did not measure phytoplankton abundance or nutrient concentrations that may have varied between ponds and could have had significant effects of environmental heterogeneity in some cases.

Temporal environmental heterogeneity had no apparent influence on the colonization rates observed for any of the three taxa. Such patterns independent of local environmental control resemble random colonization and propagule rain, where each propagule has the same likelihood of reaching a habitat patch [53], and might be most important in young communities where biotic interactions are still weak. Local biotic interaction will most likely gradually become more important as communities age [19]. Strong species sorting at intermediate dispersal rates and strong local control are frequent properties of established metacommunities [12], although biotic interactions are less important in temporary ponds like ours than in permanent ponds [23].

Connectivity measures (including colonization distance) and area were the key factors determining colonization rates at the scale of the present study, although this was only evident for cladocerans and copepods. Significant spatial patterns were detected for cladocerans and copepods, but not rotifers, and together with connectivity effects indicate dispersal limitation in the former two groups, since a spatial signal is only detectable under limited dispersal [13]. Gray & Arnott [22] also found a spatial signal in cladoceran communities in lakes recovering from acidification. The spatial signal detected in our study which to a large extent involves the southern pond group may have been strongly influenced by the higher hydrological connectivity in this area, connectivity itself being a factor that was important for copepods and cladocerans. Connectivity in our study had three main components: hydrological connectivity, whether a pond was inside or outside of the two main pond clusters (Fig. 1), and colonization distance. In our study the distance between experimental and reference ponds ranged from 170–2300 m. Reports from other studies on the effect of colonization distance are inconsistent, which could be due to the spatial scale in which studies were completed or the abundance of dispersal vectors: Cáceres and Soluk [16], and Cohen and Shurin [17] found no dispersal limitation within distances of <60 m, while [30] found an obvious limiting effect for dispersal within distances of up to 180 m.

Colonization rates are inherently related to dispersal capacity, which in the passively dispersing zooplankton largely depends on dispersal stage and dispersal vector. In all three taxa, drought-resistant dormant eggs are generally regarded as the main dispersal stage [54], although the dispersal of adult stages has also been documented [30], [55]. In cyclopoid copepods, diapause eggs are unknown and instead postembryonic stages or subitaneous eggs might be dispersed [56], [31], [54]. Wind and hydrological connectivity are vectors for all three taxa, while external or internal transport by animals will largely depend on desiccation or digestion resistance of propagules [16], [57], [29], [58], [59]. Although one might expect to find a negative relation between propagule weight and uplift by wind [60], it is unclear to what extent wind dispersal for zooplankton depends on propagule size, e.g. ephippia of cladocerans vs. rotifer eggs [61].

Which colonization patterns might be produced by different dispersal vectors? If propagules are mainly transported by animals actively selecting target habitats, directed dispersal would result. For instance, waterbirds are attracted in greater numbers and diversity to larger water surfaces [62] thus increasing the number of potential dispersal events in larger ponds or flooded areas connected to ponds. Hydrological connections directly open up a dispersal passage by which faunal exchange can occur [63], [64], but also have an indirect area effect, by creating larger targets for dispersal when ponds become part of larger flooded areas. On the other hand, for wind dispersed propagules, one could expect undirected dispersal and a homogeneous propagule rain at high dispersal rates, without a clear relationship to spatial or hydrological isolation.

In our study, a clear positive effect of habitat connectivity was seen both in cladocerans and copepods, supporting dispersal by animals and hydrological connection in these two taxa. Area was positively related to cladoceran colonization rates only. One explanation for this is a target effect (i.e. larger islands intercept a higher amount of species) applicable for any dispersal mode. An alternative explanation is that dispersal occurs mainly by birds which are important vectors for cladocerans [64], [57] and which have a clear preference for larger waterbodies. Rotifer colonization rates could not be explained by any factors measured in our study. Since we did not observe spatial limitation of dispersal, we interpret rotifers as not dispersal-limited within the scale and time frame studied, and suggest they are mainly wind dispersed and transported faster and over larger distances due to their small and more abundant propagules.

Our experimental study is the first zooplankton colonization study to be performed in a restored wetland with the spatial dimensions of natural systems. By manipulating pond size, distances between ponds and general spatial arrangement of ponds in this large-scale setup, we found evidence that zooplankton dispersal is not equal for all taxa, suggesting that specific properties of the dispersal stage and the dispersal vector may lead to differential colonization patterns. We identified important determinants of colonization rates for the three main zooplankton groups. Although the patterns observed are in part consistent with predictions of the theory of island biogeography, the underlying processes are likely to be more complex than suggested by a simple target effect. However, we are aware of the limitations of studying complex dispersal processes by employing colonization rates, which include quantitative differences caused e.g. by unsuccessful colonization events.

In metacommunity ecology theory, dispersal and colonization are crucial connecting elements which determine in part the four models presented by Leibold et al. [11]. If all members of a metacommunity do not disperse equally, this should also have important consequences for community structure of pioneer communities. Results presented here suggest that, during initial stages of community build-up, important spatial signals in the community structure will be evident. As communities mature, local factors including abiotic and biotic interactions can be expected to increase in influence over time, thus transgressing from structures predicted by the mass effect model to those predicted by a species sorting model.

## Supporting Information

Figure S1 **Aerial photograph of the northern pond cluster in February 2006.** Sampled ponds are labelled. Red circles denote the location of ponds that overspilled and were connected to an adjacent flooded area. Photo credit: Hector Garrido/EBD-CSIC.
(TIF)

Figure S2 **Magnified areas of the aerial photograph in Fig. S1 that illustrate the categorical classes used to describe the variable “degree of connection” (0 =  not connected, 1 =  weak connection to small flooded area just outside pond, 2 =  connected to shallow roadside ditch, 3 =  connected to large flooded area). Note: class 0 not shown.**
(TIF)

Figure S3 **Colonization rates of three zooplankton taxa combined (copepods, cladocerans, rotifers) measured in the sampled experimental and reference ponds throughout the study period.** Each colored line represents the colonization rate for an individual pond. Colonization rates (calculated as cumulative species richness per pond for all sample dates) were calculated for the number of days a given pond was inundated (inundation days). For more details see Materials and Methods.
(TIF)

Figure S4 **Colonization rates of copepods measured in the sampled experimental and reference ponds throughout the study period.** Each colored line represents the colonization rate for an individual pond. Colonization rates (calculated as cumulative species richness per pond for all sample dates) were calculated for the number of days a given pond was inundated (inundation days). For more details see Materials and Methods.
(TIF)

Figure S5 **Colonization rates of cladocerans measured in the sampled experimental and reference ponds throughout the study period.** Each colored line represents the colonization rate for an individual pond. Colonization rates (calculated as cumulative species richness per pond for all sample dates) were calculated for the number of days a given pond was inundated (inundation days). For more details see Materials and Methods.
(TIF)

Figure S6 **Colonization rates of rotifers measured in the sampled experimental and reference ponds throughout the study period.** Each colored line represents the colonization rate for an individual pond. Colonization rates (calculated as cumulative species richness per pond for all sample dates) were calculated for the number of days a given pond was inundated (inundation days). For more details see Materials and Methods.
(TIF)

Figure S7 **This figure shows the potential spatial relationships between the different experimental pools.** We computed Principal Coordinates of Neighbour Matrices (PCNM) or classical distance-based Moran’s Eigenvector Maps (Borcard et al. 2011) based on their geographic locations. The first row shows PCNM1-PCNM5, the second row PCNM6-PCNM10, etc. Each plot shows the PCNM values according to their geographic location, with the full squares positive values, and open squares negative values. The size of the squares is proportional to the absolute value of the PCNM score (so a large open square corresponds to a large negative value, a large closed square to a large positive value, small squares to values close to zero).
(TIF)

Table S1 **Means and SD of environmental variables measured in the sampled experimental (exp.) and reference (ref.) ponds throughout the study period.** A MANOVA performed on all listed variables, using pond type as independent variables was significant (Wilks 0.559, F_7,182_ = 20.49, p<0.001). Significant differences between ponds were only detected for DO (dissolved oxygen) concentration, DO saturation, and for pond size (Tukey’s HSD for unequal N posthoc test, p = 0.02, p = 0.04 and p<0.01, respectively).
(TIF)

Table S2 **Species list with frequency of occurrence in experimental ponds (Exp) and reference ponds (Ref) in the respective samples months.** Shaded lines show the number of species for copepods, cladocerans and rotifers encountered in experimental and reference ponds.
(TIF)

## References

[1] MacArthur RH, Wilson EO (1967). The theory of island biogeography: Princeton University Press.. 198 p.

[2] Hubbell S (2001). The unified theory of biodiversity and biogeography; Horn HS, Levin SA, editors: Princeton University Press..

[3] Buckley RC, Knedlhans SB (1986). Beachcomber biogeography - interception of dispersing propagules by islands.. J Biogeogr.

[4] Lindo Z, Winchester NN, Didham RK (2008). Nested patterns of community assembly in the colonisation of artificial canopy habitats by oribatid mites.. Oikos.

[5] McCormick PV, Pratt JR, Jenkins DG, Cairns J (1988). A comparison of protozoan, algal, and metazoan colonization of artificial substrates of differing size.. T Am Microsc Soc.

[6] Schoener TW, Spiller DA, Losos JB (2001). Natural restoration of the species-area relation for a lizard after a hurricane.. Science.

[7] Hovestadt T, Poethke HJ (2005). Dispersal and establishment: spatial patterns and species-area relationships.. Divers Distrib.

[8] Hutchinson GE (1957). Population studies - animal ecology and demography - concluding remarks.. Cold Spring Harb Sym.

[9] Rosenzweig ML (1995). Species diversity in space and time.. Species diversity in space and time: xxi+436p.

[10] Pulliam H (2000). On the relationship between niche and distribution.. Ecol Lett.

[11] Leibold MA, Holyoak M, Mouquet N, Amarasekare P, Chase JM (2004). The metacommunity concept: a framework for multi-scale community ecology.. Ecol Lett.

[12] Cottenie K (2005). Integrating environmental and spatial processes in ecological community dynamics.. Ecol Lett.

[13] Ng ISY, Carr CM, Cottenie K (2009). Hierarchical zooplankton metacommunities: distinguishing between high and limiting dispersal mechanisms.. Hydrobiologia.

[14] Bilton DT, Freeland JR, Okamura B (2001). Dispersal in Freshwater Invertebrates: Mechanisms and Consequences.. Annu Rev Ecol Syst.

[15] Bohonak AJ, Jenkins DG (2003). Ecological and evolutionary significance of dispersal by freshwater invertebrates.. Ecology Letters.

[16] Cáceres CE, Soluk DA (2002). Blowing in the wind: a field test of overland dispersal and colonization by aquatic invertebrates.. Oecologia.

[17] Cohen GM, Shurin JB (2003). Scale-dependence and mechanisms of dispersal in freshwater zooplankton.. Oikos.

[18] Cadotte MW, Mai DV, Jantz S, Collins MD, Keele M (2006). On Testing the Competition-Colonization Trade-Off in a Multispecies Assemblage.. Am Nat.

[19] Shurin JB (2000). Dispersal limitation, invasion resistance, and the structure of pond zooplankton communities.. Ecology.

[20] Sarnelle O, Knapp RA (2004). Zooplankton recovery after fish removal: Limitations of the egg bank.. Limnol Oceanogr.

[21] Louette G, De Meester L (2005). High dispersal capacity of cladoceran zooplankton in newly founded communities.. Ecology.

[22] Gray DK, Arnott SE (2011). Does dispersal limitation impact the recovery of zooplankton communities damaged by a regional stressor?. Ecol Appl.

[23] Silver CA, Vamosi SM, Bayley SE (2012). Temporary and permanent wetland macroinvertebrate communities: Phylogenetic structure through time.. Acta Oecol-Int J Ecol.

[24] Finlay BJ (2002). Global Dispersal of Free-Living Microbial Eukaryote Species.. Science.

[25] Frisch D, Moreno-Ostos E, Green AJ (2006). Species richness and distribution of copepods and cladocerans in temporary ponds of Doñana Natural Park.. Hydrobiologia 556: 327–340, DOI 310.1007/s10750–10005–11305-z.

[26] Waterkeyn A, Grillas P, Vanschoenwinkel B, Brendonck L (2008). Invertebrate community patterns in Mediterranean temporary wetlands along hydroperiod and salinity gradients.. Freshwater Biol.

[27] Jenkins DG, Buikema AL (1998). Do similar communities develop in similar sites? A test with zooplankton structure and function.. Ecol Monogr.

[28] Jenkins DG, Underwood MO (1998). Zooplankton may not disperse readily in wind, rain, or waterfowl.. Hydrobiologia.

[29] Vanschoenwinkel B, Gielen S, Seaman M, Brendonck L (2008). Any way the wind blows - frequent wind dispersal drives species sorting in ephemeral aquatic communities.. Oikos.

[30] Allen MR (2007). Measuring and modeling dispersal of adult zooplankton.. Oecologia.

[31] Frisch D, Green AJ (2007). Copepods come in first: rapid colonization of newly built temporary ponds.. Fund Appl Limnol.

[32] Vanschoenwinkel B, Waterkeyn A, Vandecaetsbeek T, Pineau O, Grillas P (2008). Dispersal of freshwater invertebrates by large terrestrial mammals: a case study with wild boar (*Sus scrofa*) in Mediterranean wetlands.. Freshwater Biol.

[33] Chase JM (2003). Community assembly: when should history matter?. Oecologia.

[34] De Meester L, Gómez A, Okamura B, Schwenk K (2002). The Monopolization Hypothesis and the dispersal–gene flow paradox in aquatic organisms.. Acta Oecol.

[35] Badosa A, Frisch D, Arechederra A, Serrano L, Green AJ (2010). Recovery of zooplankton diversity in a restored Mediterranean temporary marsh in Doñana National Park (SW Spain).. Hydrobiologia.

[36] Santamaria L, Green AJ, Diaz-Delgado R, Bravo MA, Castellanos EM, García F, Marín C (2005). Caracoles, a new laboratory for science and wetland restoration.. http://www.unesco.org/mab/publications/pdf/E_Donana.pdf.

[37] Frisch D, Arechederra A, Green AJ (2009). Recolonisation potential of zooplankton propagule banks in natural and agriculturally modified sections of a semiarid temporary stream (Doñana, Southwest Spain).. Hydrobiologia.

[38] Espinar JL, Serrano L (2009). A quantitative hydrogeomorphic approach to the classification of temporary wetlands in the Doñana National Park (SW Spain).. Aquat Ecol.

[39] Serrano L, Reina M, Martín G, Reyes I, Arechederra A (2006). The aquatic systems of Doñana (SW Spain): watersheds and frontiers.. Limnetica.

[40] Dussart B (1967). Les Copépodes des eaux continentales d’Europe occidentale. I: Calanoides & Harpacticoïdes; Boubée, editor. Paris.. 500 p.

[41] Dussart B (1969). Les Copépodes des eaux continentales d’Europe occidentale. II: Cyclopoïdes et Biologie; Boubée, editor. Paris.. 292 p.

[42] Einsle U (1993). Crustacea: Copepoda: Calanoida und Cyclopoida; Schwoerbel J, Zwick P, editors.. Stuttgart, New York: Gustav Fischer Verlag.

[43] Alonso M, editor (1996). Crustacea, Branchiopoda. Fauna Ibérica Madrid: Museo Nacional de Ciencias Naturales. CSIC.. 486 p.

[44] Koste W (1978). Rotatoria. Die Rädertiere Mitteleuropas begründet von Max Voigt. Mononongonta. (in German).. Berlin: Gebrüder Borntraeger.

[45] Jackson DA (1993). Stopping rules in principal components analysis: a comparison of heuristical and statistical approaches.. Ecology.

[46] Borcard D, Legendre P, Avois-Jacquet C, Tuomisto H (2004). Dissecting the spatial structure of ecological data at multiple scales.. Ecology.

[47] Borcard D, Gillet F, Legendre P (2011). Numerical Ecology with R: Springer.. 302 p.

[48] Legendre P, Legendre L (1998). Numerical Ecology. Amsterdam: Elsevier.. xv +853 p.

[49] Peres-Neto PR, Legendre P, Dray S, Borcard D (2006). Variation partitioning of species data matrices: estimation and comparison of fractions.. Ecology.

[50] Blanchet FG, Legendre P, Borcard D (2008). Forward selection of explanatory variables.. Ecology.

[51] Louette G, De Meester L, Declerck S (2008). Assembly of zooplankton communities in newly created ponds.. Freshwater Biol.

[52] Fahd K, Arechederra A, Florencio M, Leon D, Serrano L (2009). Copepods and branchiopods of temporary ponds in the Doñana Natural Area (SW Spain): a four-decade record (1964–2007).. Hydrobiologia.

[53] Resetarits WJ, Binckley CA, Chalcraft DR (2005). Habitat selection, species interactions, and processes of community assembly in complex landscapes - A metacommunity perspective.. Metacommunities: Spatial Dynamics and Ecological Communities.

[54] Gyllstrom M, Hansson LA (2004). Dormancy in freshwater zooplankton: Induction, termination and the importance of benthic-pelagic coupling.. Aquat Sci.

[55] Waterkeyn A, Vanschoenwinkel B, Elsen S, Anton-Pardo M, Grillas P (2010). Unintentional dispersal of aquatic invertebrates via footwear and motor vehicles in a Mediterranean wetland area.. Aquat Conserv.

[56] Bartholmé S, Samchyshyna L, Santer B, Lampert W (2005). Subitaneous eggs of freshwater copepods pass through fish guts: Survival, hatchability, and potential ecological implications.. Limnol Oceanogr.

[57] Frisch D, Green AJ, Figuerola J (2007). High dispersal capacity of a broad spectrum of aquatic invertebrates via waterbirds.. Aquat Sci.

[58] Vanschoenwinkel B, Gielen S, Vandewaerde H, Seaman M, Brendonck L (2008). Relative importance of different dispersal vectors for small aquatic invertebrates in a rock pool metacommunity.. Ecography.

[59] Brochet AL, Gauthier-Clerc M, Guillemain M, Fritz H, Waterkeyn A (2010). Field evidence of dispersal of branchiopods, ostracods and bryozoans by teal (*Anas crecca*) in the Camargue (southern France).. Hydrobiologia.

[60] Nathan R, Katul GG, Horn HS, Thomas SM, Oren R (2002). Mechanisms of long-distance dispersal of seeds by wind.. Nature.

[61] Jenkins DG, Brescacin CR, Duxbury CV, Elliott JA, Evans JA (2007). Does size matter for dispersal distance?. Global Ecol Biogeogr.

[62] Guadagnin DL, Maltchik L (2007). Habitat and landscape factors associated with neotropical waterbird occurrence and richness in wetland fragments.. Biodivers Conserv.

[63] Frisch D, Threlkeld ST (2005). Flood-mediated dispersal versus hatching: early recolonisation strategies of copepods in floodplain ponds.. Freshwater Biol.

[64] Michels E, Cottenie K, Neys L, De Gelas K, Coppin P (2001). Geographical and genetic distances among zooplankton populations in a set of interconnected ponds: a plea for using GIS modelling of the effective geographic distance.. Mol Ecol.

