# Supplementary figures and images for: Strong Spatial Influence on Colonization Rates in a Pioneer Zooplankton Metacommunity
Source: PLoS One. 2012 Jul 6;7(7):e40205. doi: 10.1371/journal.pone.0040205 (PMC3391295; doi:10.1371/journal.pone.0040205)

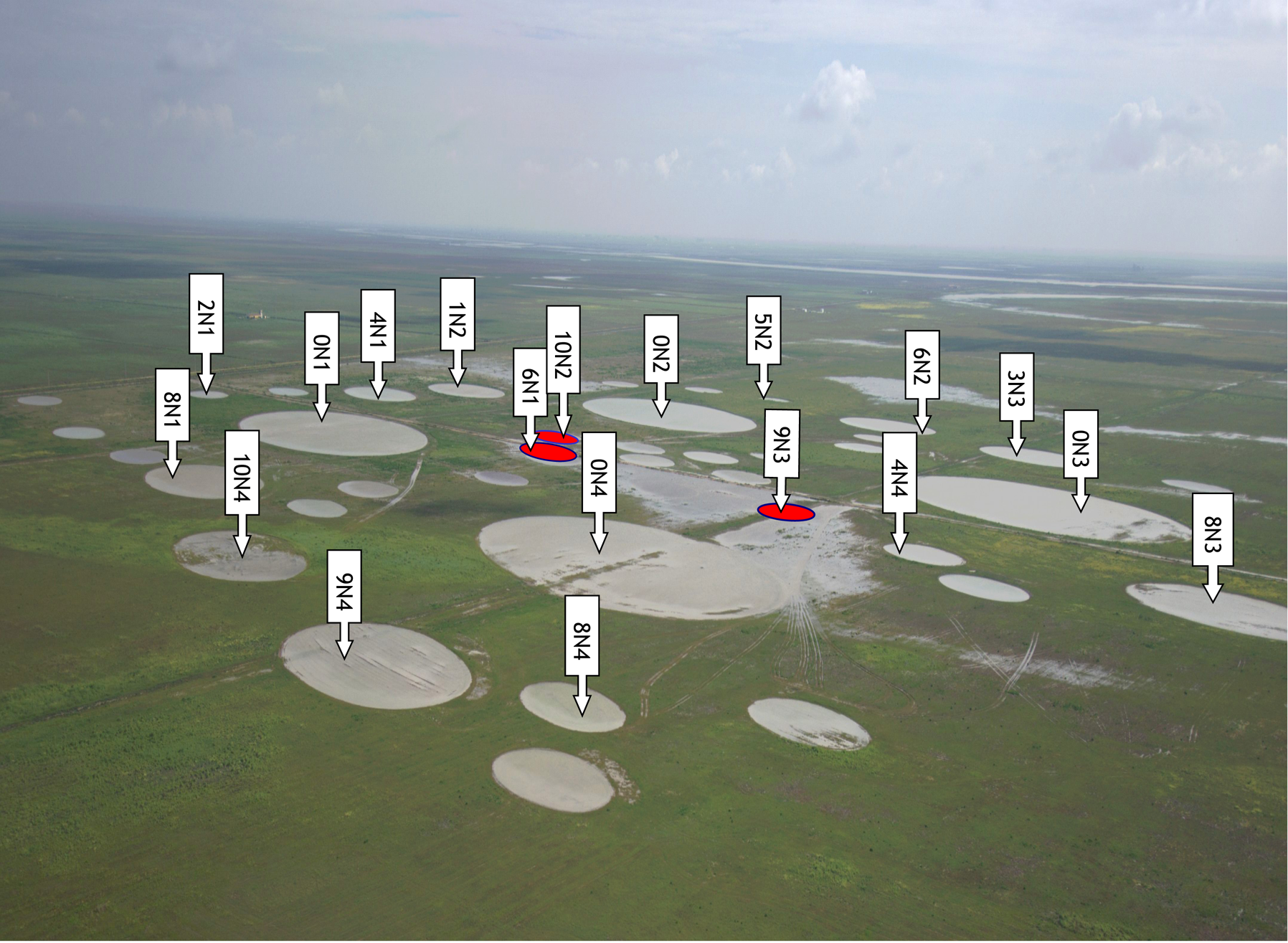

Supplement: Figure S1 — Aerial photograph of the northern pond cluster in February 2006. Sampled ponds are labelled. Red circles denote the location of ponds that overspilled and were connected to an adjacent flooded area. Photo credit: Hector Garrido/EBD-CSIC. (TIF) [file pone.0040205.s001.tif]

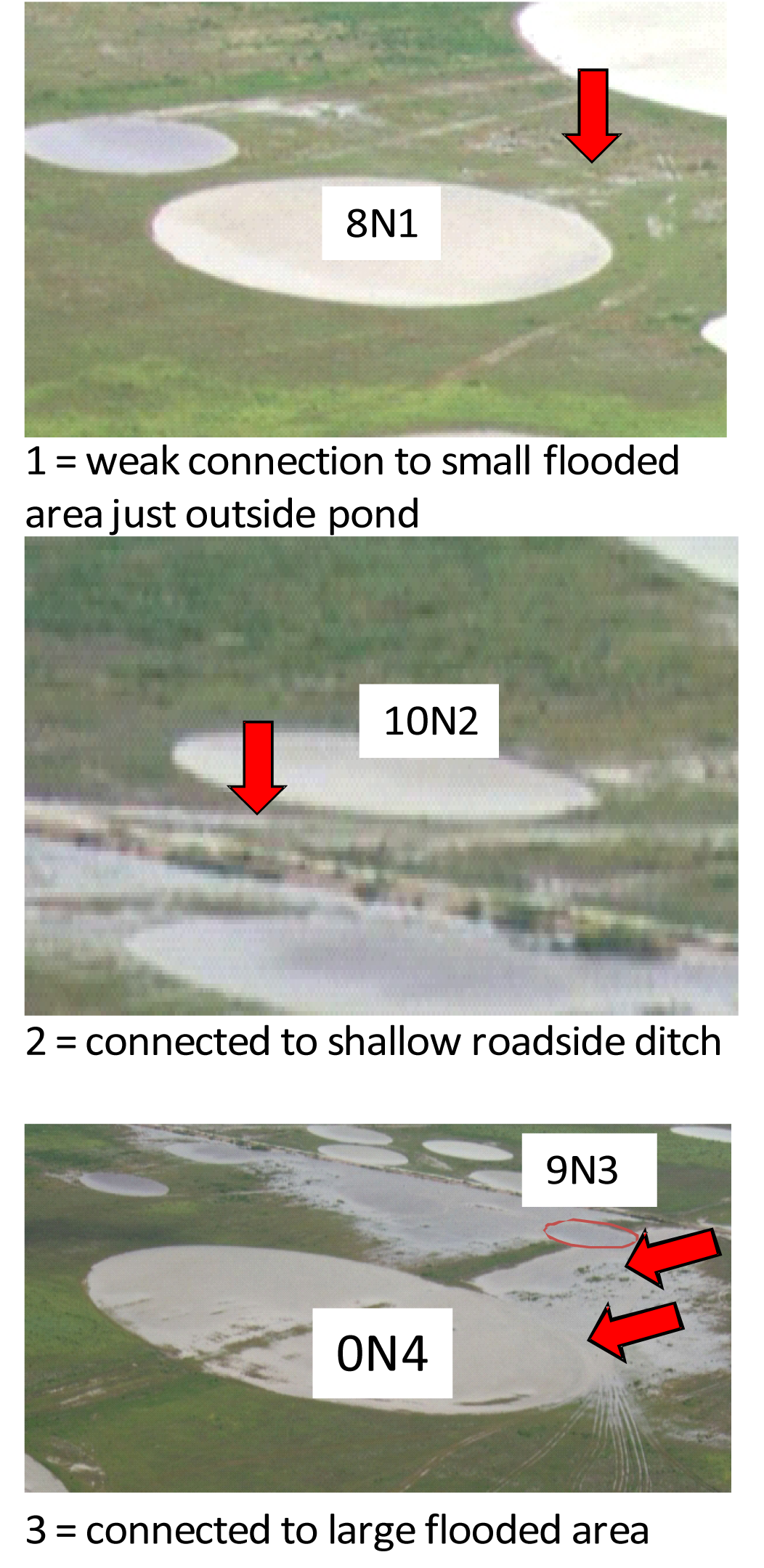

Supplement: Figure S2 — Magnified areas of the aerial photograph in Fig. S1 that illustrate the categorical classes used to describe the variable “degree of connection” (0 = not connected, 1 = weak connection to small flooded area just outside pond, 2 = connected to shallow roadside ditch, 3 = connected to large flooded area). Note: class 0 not shown. (TIF) [file pone.0040205.s002.tif]

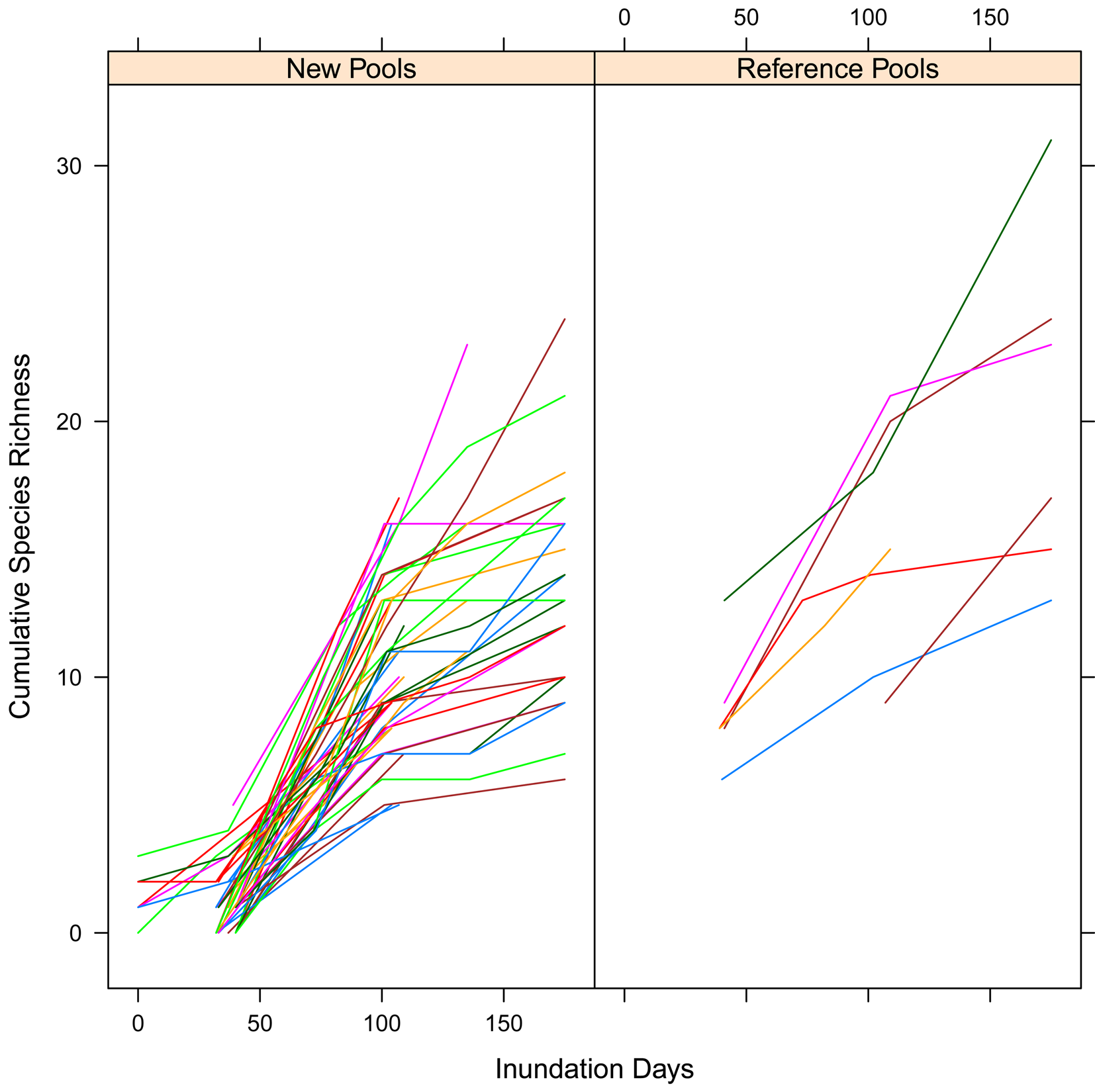

Supplement: Figure S3 — Colonization rates of three zooplankton taxa combined (copepods, cladocerans, rotifers) measured in the sampled experimental and reference ponds throughout the study period. Each colored line represents the colonization rate for an individual pond. Colonization rates (calculated as cumulative species richness per pond for all sample dates) were calculated for the number of days a given pond was inundated (inundation days). For more details see Materials and Methods. (TIF) [file pone.0040205.s003.tif]

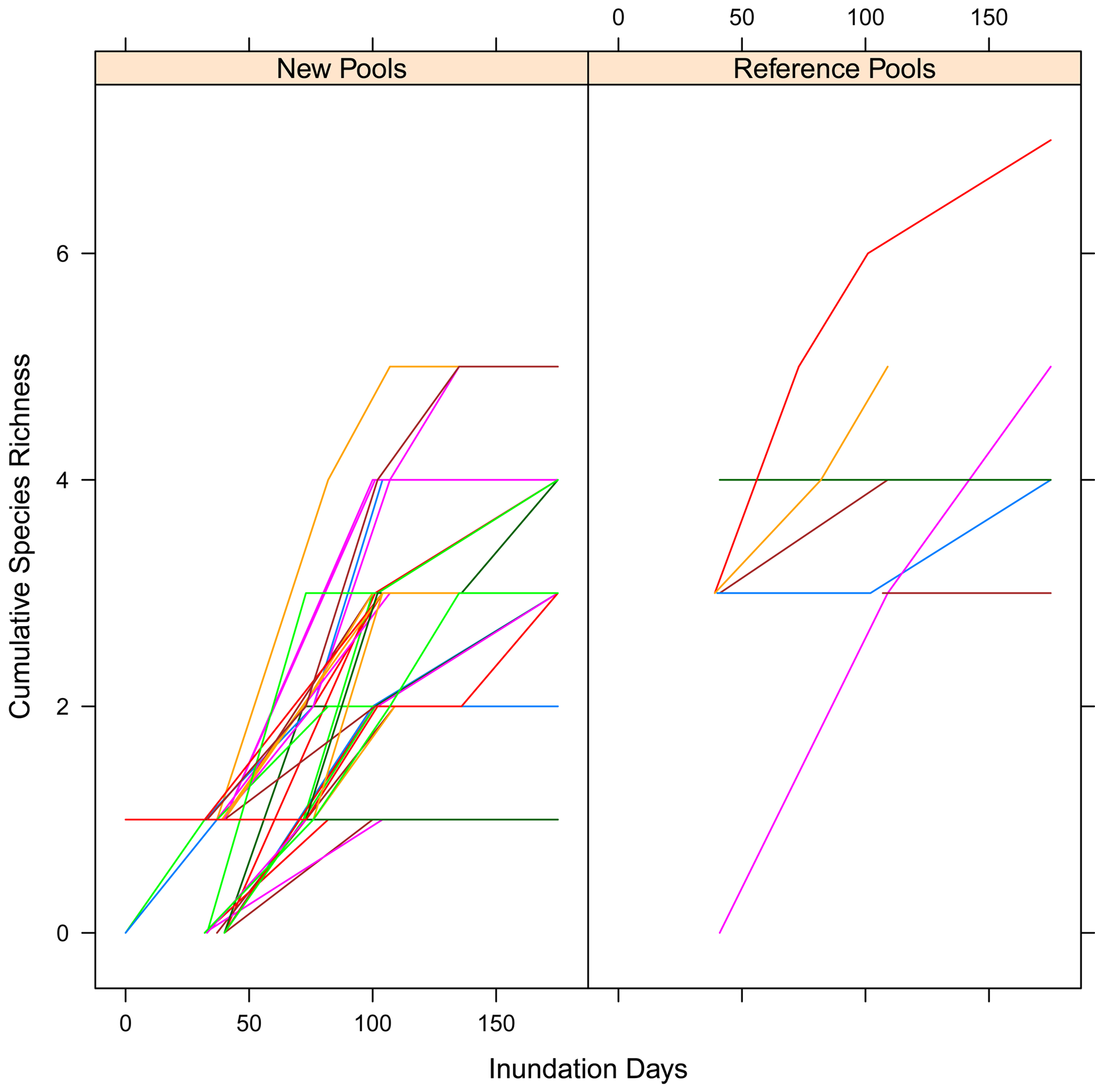

Supplement: Figure S4 — Colonization rates of copepods measured in the sampled experimental and reference ponds throughout the study period. Each colored line represents the colonization rate for an individual pond. Colonization rates (calculated as cumulative species richness per pond for all sample dates) were calculated for the number of days a given pond was inundated (inundation days). For more details see Materials and Methods. (TIF) [file pone.0040205.s004.tif]

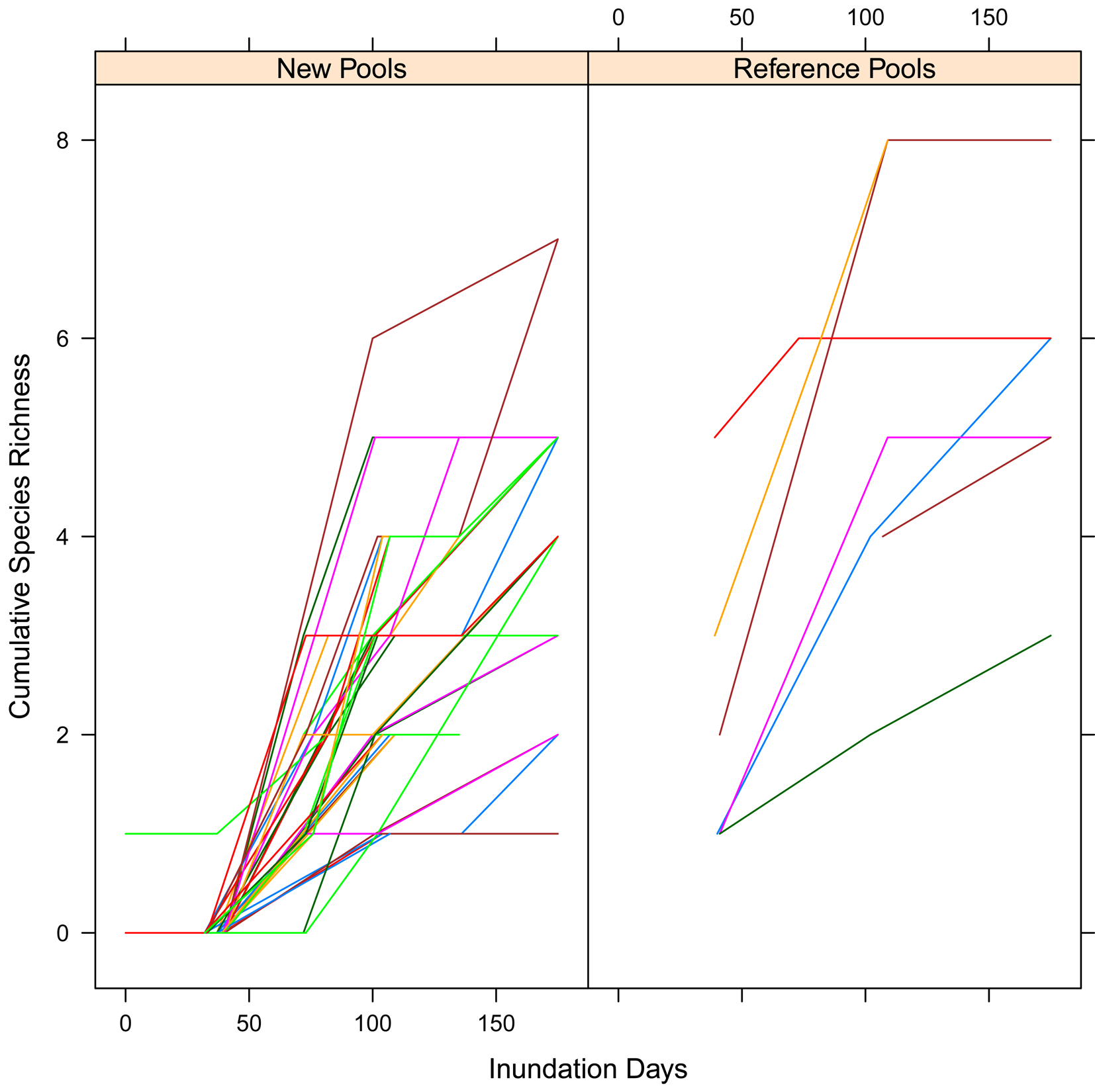

Supplement: Figure S5 — Colonization rates of cladocerans measured in the sampled experimental and reference ponds throughout the study period. Each colored line represents the colonization rate for an individual pond. Colonization rates (calculated as cumulative species richness per pond for all sample dates) were calculated for the number of days a given pond was inundated (inundation days). For more details see Materials and Methods. (TIF) [file pone.0040205.s005.tif]

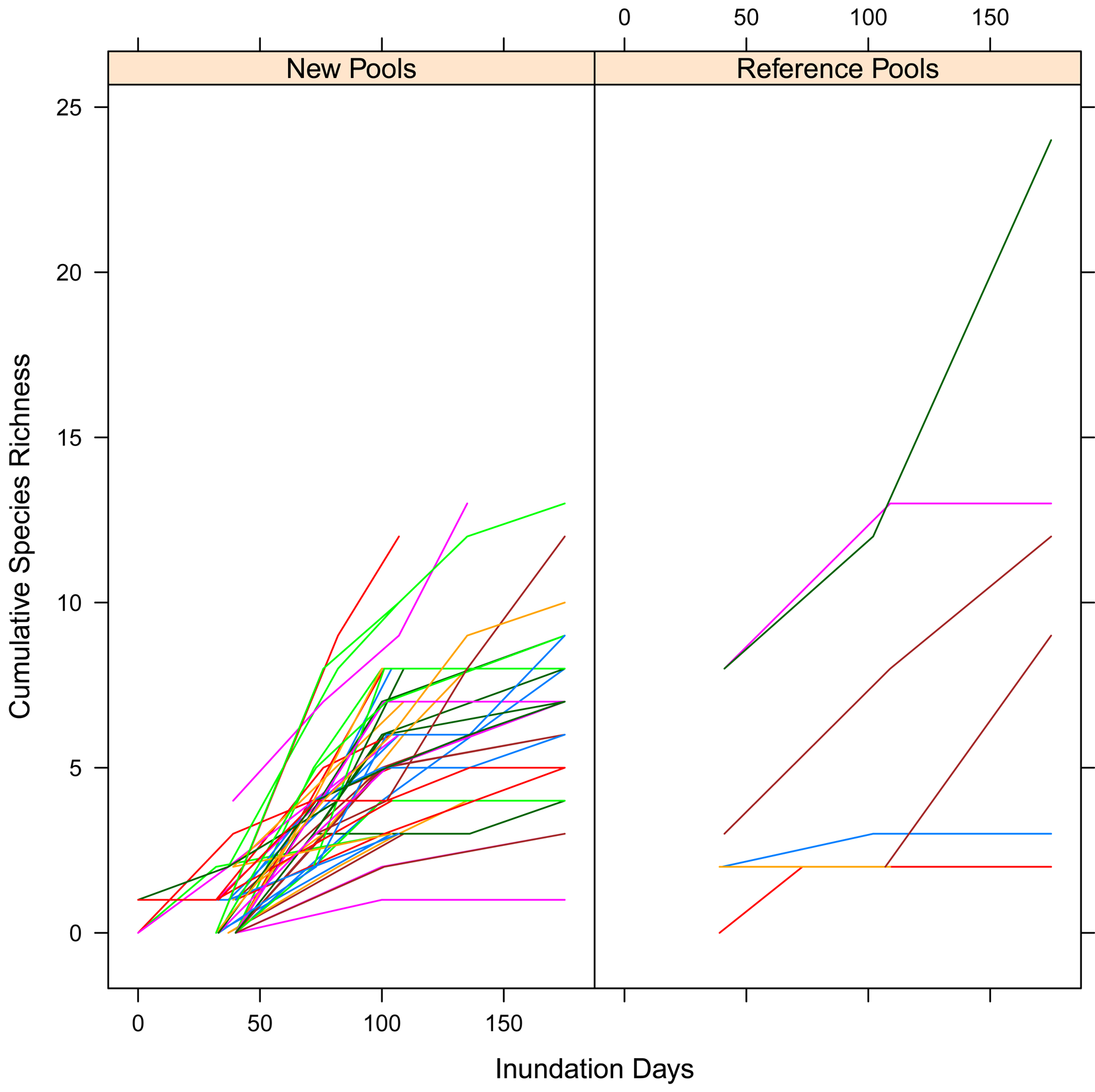

Supplement: Figure S6 — Colonization rates of rotifers measured in the sampled experimental and reference ponds throughout the study period. Each colored line represents the colonization rate for an individual pond. Colonization rates (calculated as cumulative species richness per pond for all sample dates) were calculated for the number of days a given pond was inundated (inundation days). For more details see Materials and Methods. (TIF) [file pone.0040205.s006.tif]

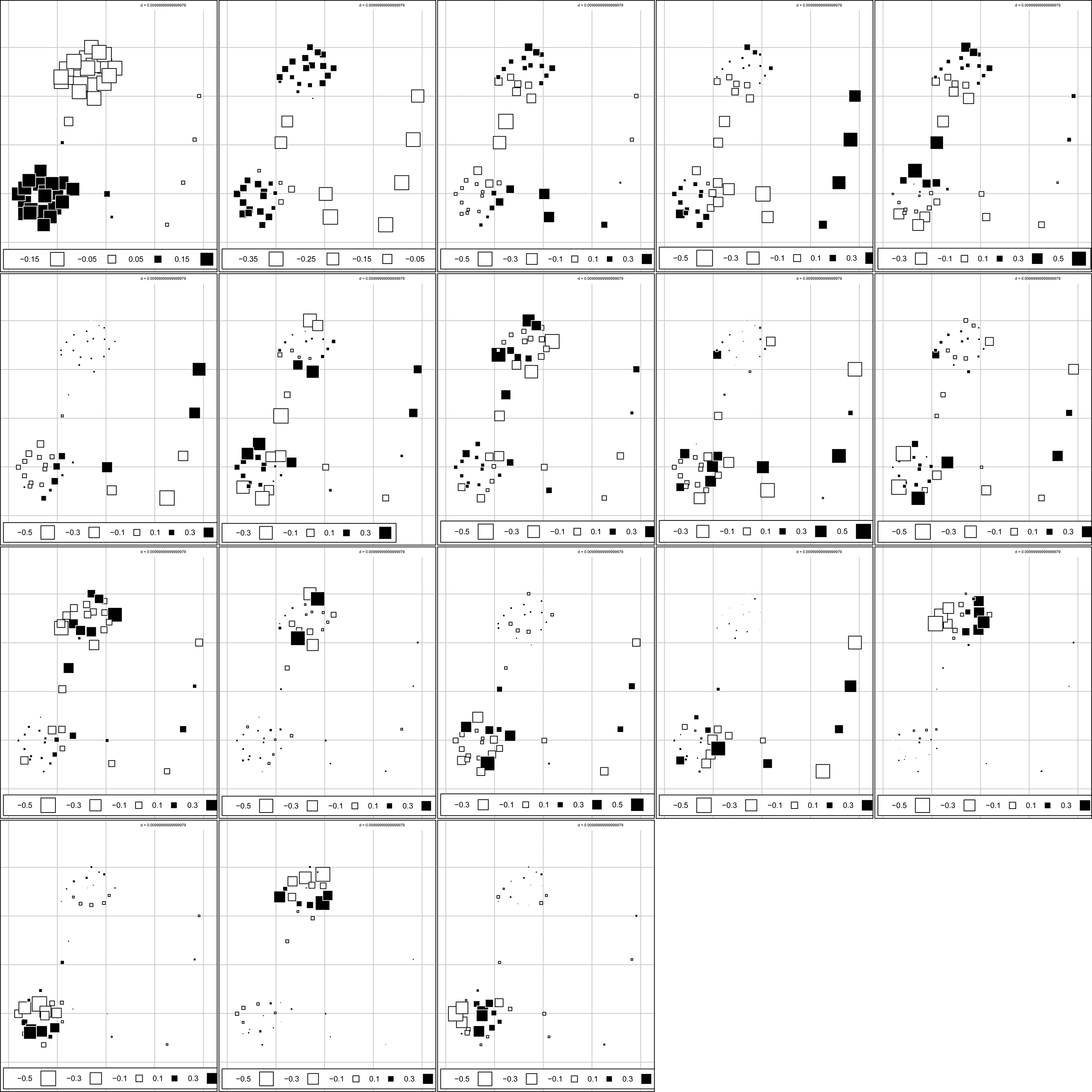

Supplement: Figure S7 — This figure shows the potential spatial relationships between the different experimental pools. We computed Principal Coordinates of Neighbour Matrices (PCNM) or classical distance-based Moran’s Eigenvector Maps (Borcard et al. 2011) based on their geographic locations. The first row shows PCNM1-PCNM5, the second row PCNM6-PCNM10, etc. Each plot shows the PCNM values according to their geographic location, with the full squares positive values, and open squares negative values. The size of the squares is proportional to the absolute value of the PCNM score (so a large open square corresponds to a large negative value, a large closed square to a large positive value, small squares to values close to zero). (TIF) [file pone.0040205.s007.tif]

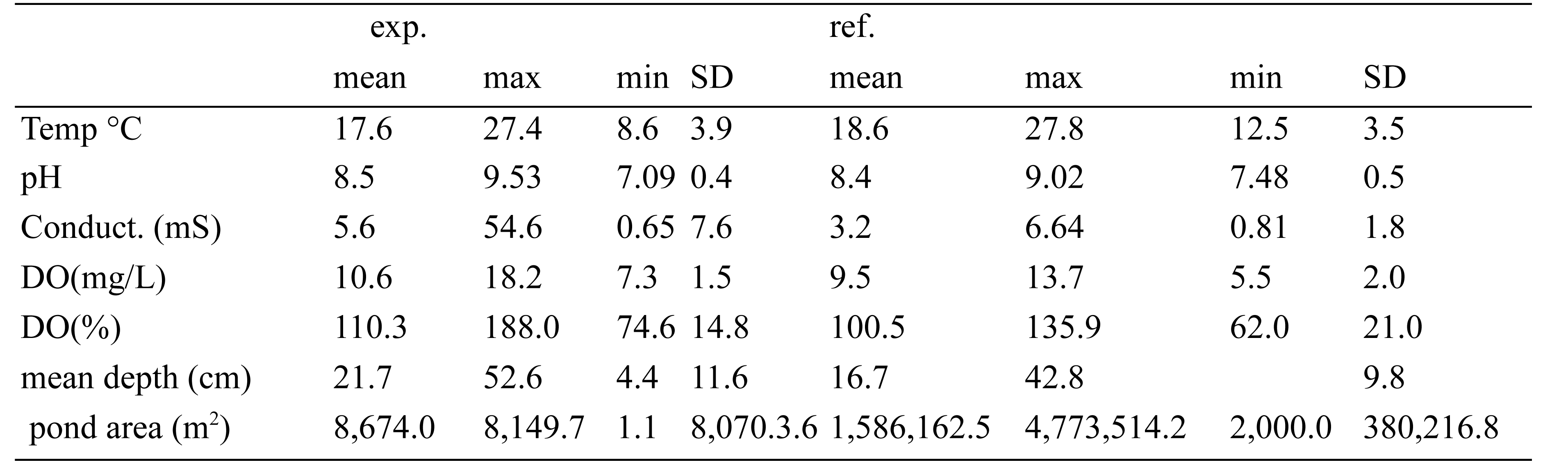

Supplement: Table S1 — Means and SD of environmental variables measured in the sampled experimental (exp.) and reference (ref.) ponds throughout the study period. A MANOVA performed on all listed variables, using pond type as independent variables was significant (Wilks 0.559, F7,182 = 20.49, p<0.001). Significant differences between ponds were only detected for DO (dissolved oxygen) concentration, DO saturation, and for pond size (Tukey’s HSD for unequal N posthoc test, p = 0.02, p = 0.04 and p<0.01, respectively). (TIF) [file pone.0040205.s008.tif]

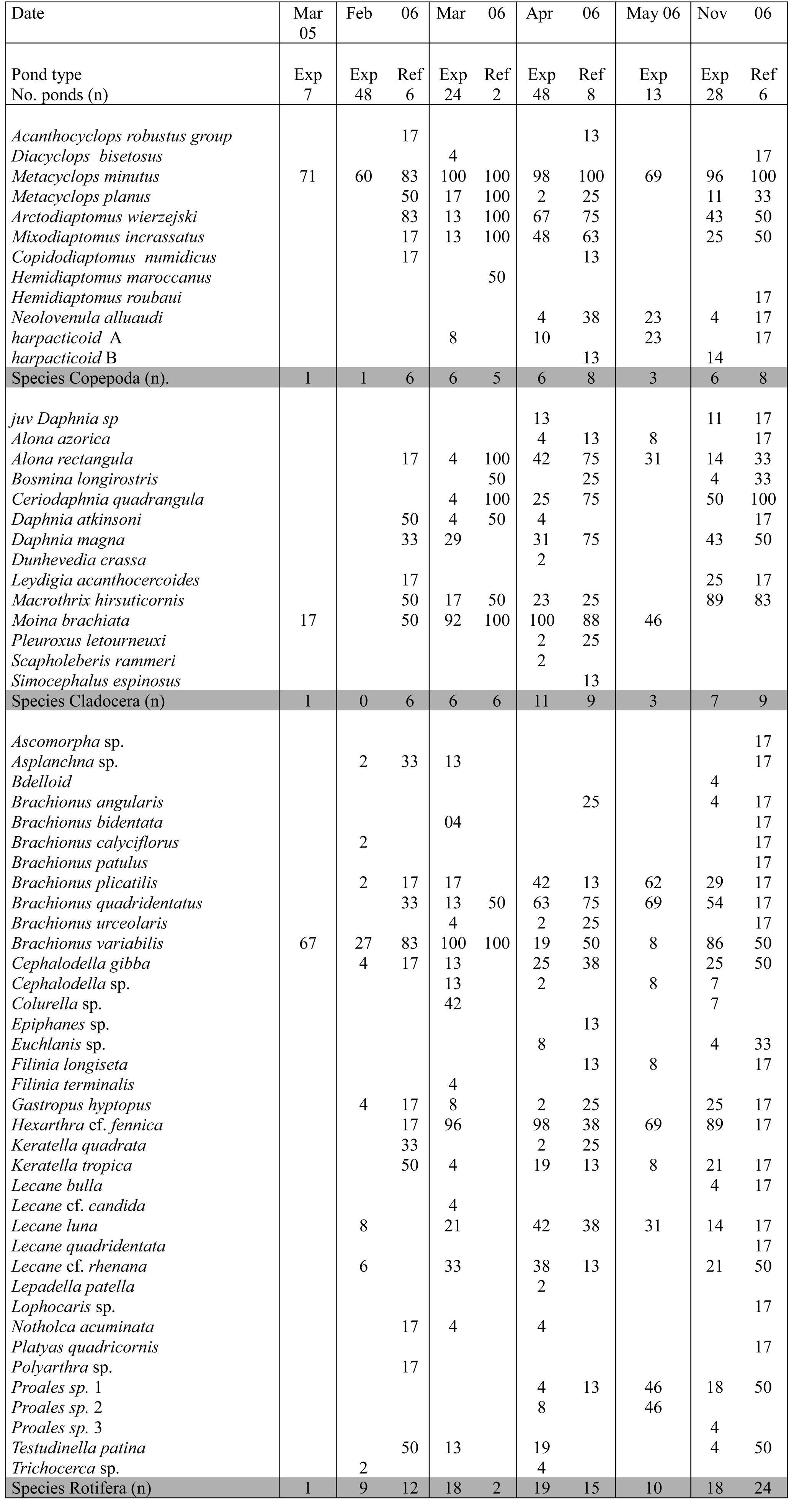

Supplement: Table S2 — Species list with frequency of occurrence in experimental ponds (Exp) and reference ponds (Ref) in the respective samples months. Shaded lines show the number of species for copepods, cladocerans and rotifers encountered in experimental and reference ponds. (TIF) [file pone.0040205.s009.tif]
